# Loci Associated with Negative Heterosis for Viability and Meat Productivity in Interspecific Sheep Hybrids

**DOI:** 10.3390/ani13010184

**Published:** 2023-01-03

**Authors:** Alexander S. Zlobin, Natalia A. Volkova, Natalia A. Zinovieva, Baylar S. Iolchiev, Vugar A. Bagirov, Pavel M. Borodin, Tatiana I. Axenovich, Yakov A. Tsepilov

**Affiliations:** 1 Kurchatov Genomic Center, Institute of Cytology and Genetics, Siberian Branch of the Russian Academy of Sciences SB RAS, 630090 Novosibirsk, Russia; 2 L.K. Ernst Federal Science Center for Animal Husbandry, 101000 Moscow, Russia; 3 Institute of Cytology and Genetics, SB RAS, 630090 Novosibirsk, Russia

**Keywords:** heterosis, overdominant model of inheritance, sheep

## Abstract

**Simple Summary:**

One of the main aims of selection is enhancing the economically important traits of animals and plants. Crossbreeding is one of the common ways to improve the economically important traits. Usually, different species of one genus are used for crossbreeding. The use of different closely related species is known as hybridization. It is expected that hybrids will have advantages over their parents in terms of economic productivity due to the effect known as heterosis. However, the phenomenon of negative heterosis exists and can induce negative impacts on different important traits, such as viability, reproductivity, productivity, etc. The exact biological mechanisms of negative heterosis remain unknown. In this study, we aimed to determine the genetic factors associated with negative heterosis in interspecific hybrids between domestic sheep (*Ovis aries*) and argali (*Ovis ammon*). We discovered one novel locus associated with viability and two novel loci associated with meat productivity. For loci associated with meat productivity, we demonstrated the effect of negative heterosis. These results may help to understand one of the possible genetic mechanisms of negative heterosis.

**Abstract:**

Negative heterosis can occur on different economically important traits, but the exact biological mechanisms of this phenomenon are still unknown. The present study focuses on determining the genetic factors associated with negative heterosis in interspecific hybrids between domestic sheep (*Ovis aries*) and argali (*Ovis ammon*). One locus (rs417431015) associated with viability and two loci (rs413302370, rs402808951) associated with meat productivity were identified. One gene (*ARAP2*) was prioritized for viability and three for meat productivity (*PDE2A, ARAP1,* and *PCDH15*). The loci associated with meat productivity were demonstrated to fit the overdominant inheritance model and could potentially be involved int negative heterosis mechanisms.

## 1. Introduction

Producing new breeds is an essential process for increasing the phenotypic indices of economically important traits, and hybridization is one of the ways to do so. Usually, different species of one genus are used for hybridization, but in some cases different closely related species are used. For example, in sheep genetics there are a few examples of hybridization of domestic sheep (*Ovis aries*) with their wild ancestors, such as the Asian mouflon (*Ovis orientalis*) [1], Pamir argali or Marco Polo sheep (*Ovis ammon polii*) [2], European mouflon (*Ovis orientalis musimon*) [3], and bighorn sheep (*Ovis canadensis*) [4]. It is expected that hybrids will have advantages over their parents in terms of economic productivity due to the effect known as heterosis, e.g., for the intraspecific hybrids of domestic sheep, the proportion of individually retained heterosis was the most important genetic factor associated with increased lamb survival [5]. Hybridization with wild ancestors is usually performed for increasing the body size, survivability, disease resistance, etc. For instance, hybrids of domestic sheep with argali showed increased lean meat production [1], while hybrids with bighorn sheep demonstrated resistance to *Mannheimia haemolytica* in the absence of vaccination [4].

Negative heterosis, on the other hand, can have negative effects on different important traits, such as viability, reproductivity, productivity, etc. Unfortunately, despite its economic importance, this phenomenon remains understudied.

In quantitative genetics there are several models of heterosis. The genes causing this phenomenon may affect traits in three different ways: dominance (attributes heterosis to the canceling of deleterious or inferior recessive alleles contributed by one parent, by beneficial or superior dominant alleles contributed by the other parent in the heterozygous genotypes at different loci) [6,7,8], overdominance (attributes heterosis to the superior fitness of heterozygous genotypes over homozygous genotypes at a single locus) [8,9], and epistasis (contribution of epistatic interactions between non-allelic genes) [10,11]. In the field of plant genetics, different studies favor one mechanism over another [12,13,14,15].

As for domestic animals, the majority of studies on negative heterosis and its possible genetic and cytogenetic mechanisms have been conducted on chickens [16,17]. These studies have found that non-additive genes, their related oxidative phosphorylation, and the difficulties in homology matching between the DNA sequences of genetically divergent breeds might lead to negative heterosis. As for other domestic animals, there are only several publications, but they do not provide sufficient information about the possible mechanisms of this phenomenon [18,19]. In any case, to claim that a given associated locus is involved in heterosis, it is not enough to simply prove that it is associated with the trait—the locus should also have a non-additive inheritance model with negative overdominance. Such a model is a classic example of negative heterosis, when heterozygotes show inferior performance compared to homozygotes.

Previously, our group focused on obtaining a population of hybrids between domestic sheep (*Ovis aries*) and argali (*Ovis ammon*) [20]. In this study, a strong negative heterosis was observed for interspecific hybrids relative to their diagonal body length and their chest width and depth. In addition, many hybrid lambs died in the first 3 months after birth, reflecting negative heterosis with respect to viability. Additionally, considering the fact that the genetic and molecular mechanisms of negative heterosis in sheep are unknown, the focus of the present study was on determining the genetic factors associated with negative heterosis in interspecific hybrids between domestic sheep (*Ovis aries*) and argali (*Ovis ammon*).

## 2. Materials and Methods

### 2.1. Animals

A total of 89 hybrids of F1 Romanovskaya × argali backcross were analyzed. Handling and breeding of the Russian sheep population followed the protocols approved by the Ethics Committee on Animal Care and Use of the Institute of Cytology and Genetics (approval # 45/2 of 10 January 2019).

### 2.2. Genotype Data

The animals were genotyped using the Ovine Infinium^®^ HD SNP BeadChip containing 603,350 (HD) SNPs (Illumina Inc., San Diego, CA, USA). The alleles were coded using the Illumina A/B scheme. Quality control of the hybrid genotypes was performed using information about four additional groups of animals (argali—10 animals; Katahdin—14; Romanovskaya—18; F1 hybrids of Romanovskaya and Katahdin—14). Earlier, some SNPs had been noted to have a very low quality of genotypes in each of these groups. These SNPs were removed from the analyzed hybrid genotypes to prevent methodological errors. Then, a standard quality control procedure was carried out, according to the following SNP and sample inclusion criteria: SNP and sample call rate > 0.98; sample heterozygosity within ±3 standard deviation ranges; and minor allele frequency (MAF) > 0.01. As a result, 87 animals and 446,790 SNPs passed the quality control.

To calculate a kinship matrix, a subset of the pruned SNPs that were in approximate linkage equilibrium with one another was generated using such options as maf 0.05—indep-pairwise 500 kb 10.3 from PLINK 1.9 [21]. In total, 46,471 pruned SNPs were generated.

The kinship matrix was calculated using the IBs function from the R package GenABEL [22].

### 2.3. Phenotype Data

Eleven carcass traits (see Supplementary Table S1) were measured four times—after birth, at 6, 42, 90, and 180 days, identifying 44 phenotypes in total. Based on these traits, four integral and holistic indices (see Supplementary Table S2) were calculated that related to meat productivity in sheep and cows [23,24]. These four indices and body mass were only used in relation to the 42-day timepoint as the latest one to reasonably affect the animals’ survival rate. The missing phenotypes were imputed using the R package PHENIX [25]. For the imputation, all 11 original traits for 6 and 42 days after birth were applied.

If an animal had no phenotypic information starting from a given time, it was considered to be deceased by that timepoint. The numbers of living animals for each timepoint are presented in Table 1.

### 2.4. Genome-Wide Association Study (GWAS)

For both viability and meat productivity traits, an association test with two degrees of freedom was carried out, which did not restrict the locus inheritance model. The probable inheritance model was assessed by either association boxplots or by the lowest-association *p*-value for one-degree association tests for additive, recessive, dominant, and overdominant models. The test was applicable only for the SNPs with all three genotypic classes available. If an SNP had only two genotypic classes, a one-degree-of-freedom test without inheritance model inference was applied.

#### 2.4.1. Viability GWAS

We used the Cox regression model from the R package SURVIVAL. The likelihood ratio test (LRT) was applied for estimating association *p*-values based on the following procedure. The null model (H0) was set as follows:

Survivability~sex + number of fetus + Genetic principal component 1 + Genetic principal component 2(1)

where survivability is the status of the animal (i.e., alive or dead) and when it died (see Section 2.3 for more details), and the 4 covariates (sex, number of fetuses, and genetic principal components 1 and 2) are based on the variance-standardized relationship matrix. For the alternative model (H1), SNP genotype was added as a factor. The association statistic was calculated as follows:

−2[ln(L(H0)) − ln(L(H1))],(2)

where L( ) is the corresponding model’s likelihood. The resulting statistics were distributed as chi-squared with one degree of freedom in the event that the SNP model had only two genotypic classes, or with two degrees of freedom in the event that it had all three genotypic classes. The *p*-value significance threshold was determined as follows:

0.05/Nindep snps = 1.08 × 10^−6^ (3)

where Nindep snps = 46,271 SNPs remaining after pruning.

For a locus that passed the threshold, Cox regression using the mlreg function from the R package GenABEL was applied. This analysis was conducted for the four inheritance models (additive, dominant, recessive, and overdominant).

#### 2.4.2. GWAS of the Four Indices and Mass

For this study, a mixed linear regression model was applied. The GRAMMAR-Gamma method was used to correct the phenotypes in relation to their relatedness structure [26]. To perform this correction, an identity-by-state (IBS) matrix was calculated as described above. Sex, number of fetuses, the last time the phenotype information was available, the two first genetic principal components, and SNPs associated with viability were considered as covariates. The LRT was applied to the resulting residuals as described above. The null model was calculated via linear regression as follows:

Phenotype~1(4)

and the alternative model was defined as follows:

phenotype~SNP(5)

where the SNP was determined as a factor. The *p*-value significance threshold value was set as follows:

0.05/(4 × Nindep snps) = 2.7 × 10^−7^ (6)

where Nindep snps = 46,271 SNPs remaining after pruning, and 4 is the number of independent traits used in the analysis.

The associated loci were defined as regions within ±500 kb around the SNPs most significantly associated with the trait (lead SNPs). 

A locus was defined as new if it was not present on the list of known SNPs and genes from our previously published database of the QTLs and genes associated with meat productivity in sheep [27]. This database includes information from 24 papers published between 2013 and 2020.

Inflation factors for five traits are presented in Supplementary Table S3.

### 2.5. Functional Annotation

The Ensembl Variant Effect Predictor (VEP) was applied to analyze the potential effects of the SNPs and indels that were in high linkage disequilibrium (LD; r2 > 0.7) with the lead SNPs. The analysis was performed using the software available online [28].

### 2.6. Estimation of Heritability

For estimation of additive heritability, we used GREML analysis from GCTA software [29]. Sex, number of fetuses, the last time the phenotype information was available, the two first genetic principal components, and SNPs associated with viability were used as covariates.

### 2.7. Replication Analysis

For replication analysis, different types of hybrids of the Romanovskaya breed and Katahdin breed with argali and mouflon (35 animals in total) were used [30]. We performed the same protocol for GWAS as described above. The threshold for replication was set to

0.05/(4 × 2) = 0.00625(7)

where 4 is the number of independent traits and 2 is the number of discovered SNPs from GWAS of the four indices and mass.

## 3. Results

### 3.1. Viability GWAS

**Figure 1:**
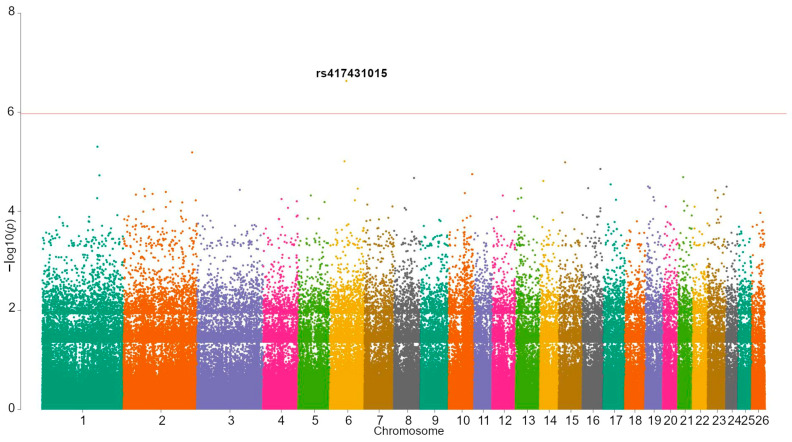
Manhattan plot of the viability GWAS. The solid red line represent a significance threshold of 1.08 × 10^−6^.

The resulting inflation factor for the GWAS was 1.071. One locus significantly associated with viability (Table 2) was found. The study’s Manhattan plot is presented in Figure 1, and the regional association plot is shown in Supplementary Figure S1. Since viability is not a quantitative trait, the association boxplots were uninformative. For that reason, we could only speculate about the locus’s inheritance model based on the association results for the four models investigated (additive, dominant, recessive, and overdominant). The lowest *p*-value (Supplementary Table S7) was observed for the additive model, with a *p*-value of 4.6 × 10^−3^, and the G-allele estimated effect was 0.051.

### 3.2. GWAS of Four Indices and Mass

**Figure 2:**
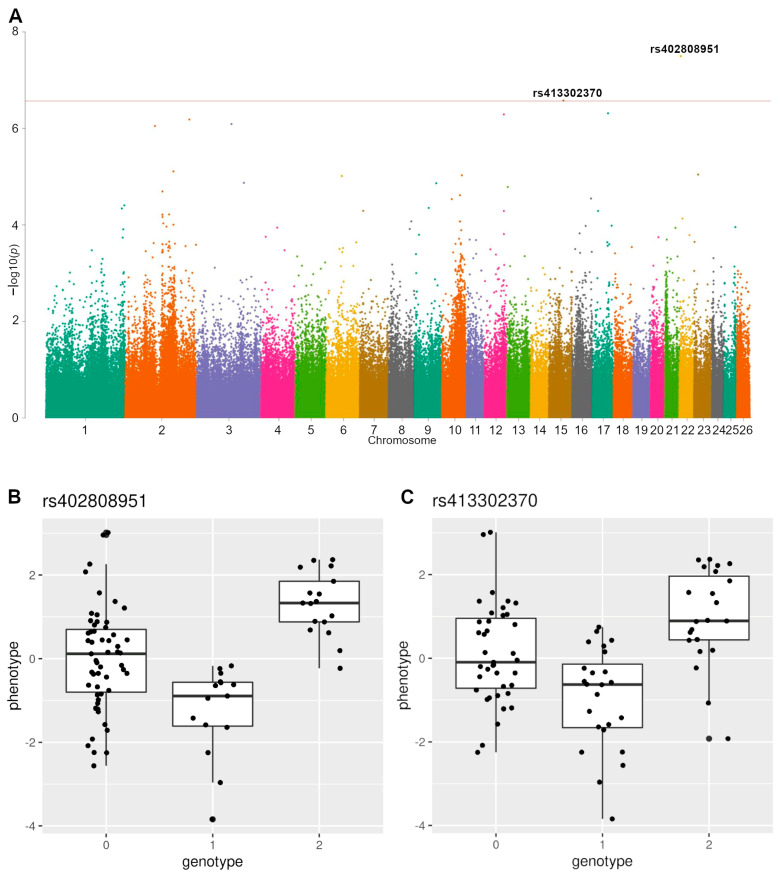
(**A**) Manhattan plot of compactness index. The solid red line represent a significance threshold of 2.7 × 10^−7^.(**B**) Boxplot for rs402808951. (**C**) Boxplot for rs413302370.

The GWAS’s results are presented in Table 1. Two loci associated with the compactness index were detected; joint Manhattan plots for the compactness index and the boxplots for the two loci can be seen in Figure 2. Manhattan plots for four other traits are presented in Supplementary Figures S2–S5. Regional association plots for the two loci can be found in Supplementary Figures S6 and S7. As the boxplots show, both variants fit a strong overdominant inheritance model. The estimated additive heritability for the traits varied from 0.46 to 0.85, with the length index having the highest heritability (see Supplementary Table S4).

### 3.3. Functional Annotation

Detailed information on the functional annotation is presented in Supplementary Tables S5 and S6. Two intron variants (rs417431015 and rs402808951) were discovered for the viability and compactness indices, respectively. The SNP of rs413302370 was located in the intergenic region.

For the locus tagged by rs417431015, associated with viability, the nearest gene *ARAP2* was prioritized. This gene is involved in a pathway that controls focal adhesion through other signal proteins [31]. It is also involved in the integrin α5β1 traffic pathway [32]. *ARAP2* influences sphingolipid metabolism through glucosylceramide synthase [33]. Furthermore, it is associated with carcass traits in Santa Ines sheep [34], loin strength in Chinese Holstein cows [35], and carcass traits in Korean cattle [36].

As for the two loci associated with meat productivity (rs413302370 and rs402808951), three genes (*PDE2A* and *ARAP1* for locus rs413302370 and *PCDH15* for locus rs402808951) were prioritized. *PDE2A* encodes the phosphodiesterase 2A protein, and its mutations are associated with movement disorders in humans, such as chorea [37] and paroxysmal dyskinesia [38]. *ARAP1* is involved in the regulation of membrane trafficking and actin cytoskeleton reorganization through activation of Arf and Rho GTPases [39]. In addition, *ARAP1* is known as a common regulatory variant for type 2 diabetes in humans [40]. Mutations in the *PCDH15* gene cause Usher syndrome type 1F in humans [41].

## 4. Discussion

In the present study, three genetic variants associated with productivity traits in sheep hybrids were found, and four genes were prioritized in these loci. Locus rs417431015, associated with viability, had quite a significant effect on the given sample size. Using the variance approximation for the quantitative traits, written as Z^2^/N, we estimated the viability variance for this locus to be about 29%. Since the most probable inheritance model for this locus was an additive one, it could not be involved in negative heterosis. For this locus, the *ARAP2* gene involved in focal adhesion was prioritized. In addition, this gene is associated with meat productivity and carcass traits in cattle and sheep. Thus, additional investigation is required to clarify the mechanism of mutation in the *ARAP2* gene, which may lead to death in interspecific sheep hybrids.

For the two loci associated with the compactness traits, a good fit to the strong overdominant inheritance model was observed. Their variances were relatively high as well, being 30.4% and 35.2% for rs413302370 and rs40280895, respectively. Interestingly, we found associations only for the compactness index, while three out four other traits demonstrated higher estimated additive heritability (length index, mass, and cumulative index). This may be related to the observed non-additive effects of SNPs associated with the compactness index and the phenomenon of negative heterosis. The overdominant model indicated the classic pattern of a genotype associated with a trait affected by heterosis, i.e., the heterozygotes showed substantially inferior performance compared to the homozygotes (Figure 2B,C). The effective allele frequencies were significantly different between the parental breeds, which also supports the hypothesis that these loci were involved in negative heterosis. Moreover, these results confirm our previous observations of negative heterosis manifestations in relation to the diagonal body length and the chest depth and width, due to the associations demonstrated for the compactness index to comprise the diagonal body length and chest girth.

Unfortunately, there are only a few examples of studies of interspecific sheep hybrids in the literature. To the best of our knowledge, there have been no studies describing the phenomenon of negative heterosis in interspecific hybrids. Our results showing negative heterosis contradict the findings of a previous study that showed increased lean meat production for interspecific sheep hybrids with Asian mouflon [1], which can be explained by the fact that different domestic sheep breeds were used for crossbreeding in the experiments. Additionally, in our study, a different set of carcass traits was used. However, in another study where domestic sheep were crossed with European mouflon (*Ovis orientalis musimon*), the hybrids had less weight than domestic sheep [3]. Our results overlap with those of a study of interspecific sheep hybrids, where domestic sheep were crossed with Pamir argali or Marco Polo sheep (*Ovis ammon polii*) [2]. In that study, the authors prioritized *PCDH10* as a candidate gene associated with body weight traits. In our study, we prioritized the *PCDH15* gene for the compactness index, which belongs to the same protein family as *PCDH10*. Further investigations of these proteins are needed to understand the possible mechanisms of influence on meat productivity traits.

It should be noted that there have been a few sheep studies where heterosis was shown and measured. The effects of direct heterosis (i.e., due to a lamb being crossbred) were small for an Australian population including more than 1 million animals [42]. In another study, a large direct and maternal effect of heterosis (due to the lambs’ dams being crossbred) was estimated for a U.S. sheep population [43]. In addition, the effect of positive heterosis was shown in a study crossing indigenous fat-tailed and commercial sheep breeds [44]. These studies show a possible positive heterosis in sheep and, in combination with our results, may help to make interspecific crosses between domestic sheep and their wild relatives more successful in terms of increasing their economically important traits.

It is noteworthy that for both viability and meat productivity the genes from one protein family (*ARAP1* and *ARAP2*) were prioritized. Both of these genes are involved in membrane transport: *ARAP1* regulates the cell-specific trafficking of a receptor protein involved in apoptosis, while *ARAP2* encodes a protein that is associated with focal adhesions and functions downstream of RhoA to regulate focal adhesion dynamics. Presumably, there is a gene network including these genes, but additional investigation into interspecific sheep hybrids is required to clarify this issue.

F2 backcrosses are preferable to observe all three genotypic classes for each SNP; it is clear that the studied population did not have the most optimal design for heterosis investigation, since it consisted of F1 backcrosses and their mother breed (Romanovskaya). For that reason, we do not expect that our findings could be replicated using other populations, considering that the possible heterosis mechanisms could be unique for each hybrid population. We performed replication analysis using other types of hybrids (35 animals in total). No SNPs reached the significance threshold of replication.

Better understanding of the genetic causes of negative heterosis in different domestic animals may help to improve breeding results. Knowledge of “proper” heterosis-associated allele combinations may help us to develop efficient marker-assisted systems that can be applied for breeding purposes.

## 5. Conclusions

In summary, the findings of the present study suggest two possible genetic markers (with three prioritized genes) that could potentially be involved in negative heterosis mechanisms in interspecific hybrids between domestic sheep and argali. In addition, we discovered one novel locus that was associated with viability. Further investigation is needed to clarify the possible molecular role of the discovered genes in negative heterosis. The results of the present study can help researchers and breeders in futures studies to create new interspecific sheep hybrid populations that are more successful in terms of economically important traits, as well as to develop efficient marker-assisted systems for breeding purposes.

## Figures and Tables

**Table 1:** Numbers of living animals for each timepoint.

Days after Birth	Number of Alive Animals
6	87
42	83
90	58
180	7

**Table 2:** Loci significantly associated with viability and compactness index.

SNP	CHR	POS	*p*	A1/A2	AF	P.11	P.12	P.22	AF Romanovskaya	AF Argali	Nearest Gene	Trait
rs417431015	6	55,587,508	2.33 × 10^−7^	G/A	0.368	28	54	5	0.5	0.6	*ARAP2*	Viability
rs413302370	15	50,414,913	2.65 × 10^−7^	A/G	0.407	39	24	23	0.667	1	*PDE2A* *ARAP1*	Compactness index
rs402808951	22	4,457,667	3.18 × 10^−8^	A/G	0.282	55	15	17	0.278	1	*PCDH15*	Compactness index

SNP—Single nucleotide polymorphism. CHR—Chromosome. POS—Position. *p*—*p*-value. AF—Frequency of A2 allele. P.11—Number of animals with two A1 alleles. P.12—Number of animals with A1 and A2 alleles. P.22—Number of animals with two A2 alleles. AF Romanovskaya—A2 frequency counted in 18 Romanovskaya sheep. AF Argali—A2 frequency counted in 10 argali (*Ovis ammon*).

## Data Availability

The data used in this study is the property of L.K. Ernst Federal Science Center for Animal Husbandry, Russia and is therefore not publicly available. The GWAS summary statistics from this study are available in the GWAS-MAP|ovis platform https://pheligeovis.icgbio.ru.

## Supplementary Materials

The following supporting information can be downloaded at: https://www.mdpi.com/article/10.3390/ani13010184/s1, Figure S1: The regional association plot for rs417431015; Figure S2: The Manhattan plot for shortlegged index; Figure S3: The Manhattan plot for length index; Figure S4: The Manhattan plot for mass; Figure S5: The Manhattan plot for cumulative index; Figure S6: The regional association plot for rs413302370; Figure S7: The regional association plot for rs402808951; Table S1: Original measured traits; Table S2: Definition of indices; Table S3: Lambda for each trait’s GWAS; Table S4: Estimated heritability for each trait’s GWAS; Table S5: Detailed results of VEP analysis for SNPs associated with viability; Table S6: Detailed results of VEP analysis for GWAS SNPs; Table S7: *p*-Values for different models of inheritance for the SNP rs417431015.

## References

[1] Jueken A., Hubdar A.K., Yiming S., Li Q.F., Xie Z. (2011). Comparative Analysis on the Performance of the Hybrid Offspring of Wild Argali and Bashibay Sheep. Afr. J. Biotechnol..

[2] Li X., He S.-G., Li W.-R., Luo L.-Y., Yan Z., Mo D.-X., Wan X., Lv F.-H., Yang J., Xu Y.-X. (2022). Genomic analyses of Pamir argali, Tibetan sheep, and their hybrids provide insights into chromosome evolution, phenotypic variation, and germplasm innovation. Genome Res..

[3] Mavrogenis A.P., Herzogi A. (1990). Interspecific Hybridization of the Cyprus Mouflon (Agrinon) with Domestic Sheep. Anim. Genet. Resour. Inf..

[4] Subramaniam R., Shanthalingam S., Bavananthasivam J., Kugadas A., Raghavan B., Batra S.A., Herndon C.N., Rodriguez J., Tibary A., Nelson D. (2014). Bighorn Sheep × Domestic Sheep Hybrids Survive Mannheimia Haemolytica Challenge in the Absence of Vaccination. Vet. Microbiol..

[5] Ferreira V.C., Rosa G.J.M., Berger Y.M., Thomas D.L. (2015). Survival in Crossbred Lambs: Breed and Heterosis Effects. J. Anim. Sci..

[6] Lippman Z.B., Zamir D. (2007). Heterosis: Revisiting the Magic. Trends Genet..

[7] Jones D.F. (1917). Dominance of Linked Factors as a Means of Accounting for Heterosis. Proc. Natl. Acad. Sci. USA.

[8] Li L., Lu K., Chen Z., Mu T., Hu Z., Li X. (2008). Dominance, Overdominance and Epistasis Condition the Heterosis in Two Heterotic Rice Hybrids. Genetics.

[9] Zhou G., Chen Y., Yao W., Zhang C., Xie W., Hua J., Xing Y., Xiao J., Zhang Q. (2012). Genetic Composition of Yield Heterosis in an Elite Rice Hybrid. Proc. Natl. Acad. Sci. USA.

[10] Yu S.B., Li J.X., Xu C.G., Tan Y.F., Gao Y.J., Li X.H., Zhang Q., Maroof M.A.S. (1997). Importance of epistasis as the genetic basis of heterosis in an elite rice hybrid. Proc. Natl. Acad. Sci. USA.

[11] Schnell F.W., Cockerham C.C. (1992). Multiplicative vs. Arbitrary Gene Action in Heterosis. Genetics.

[12] Melchinger A.E., Utz H.F., Piepho H.P., Zeng Z.B., Schön C.C. (2007). The Role of Epistasis in the Manifestation of Heterosis: A Systems-Oriented Approach. Genetics.

[13] Boeven P.H.G., Zhao Y., Thorwarth P., Liu F., Maurer H.P., Gils M., Schachschneider R., Schacht J., Ebmeyer E., Kazman E. (2020). Negative Dominance and Dominance-by-Dominance Epistatic Effects Reduce Grain-Yield Heterosis in Wide Crosses in Wheat. Sci. Adv..

[14] Luo L.J., Li Z.K., Mei H.W., Shu Q.Y., Tabien R., Zhong D.B., Ying C.S., Stansel J.W., Khush G.S., Paterson A.H. (2001). Overdominant Epistatic Loci Are the Primary Genetic Basis of Inbreeding Depression and Heterosis in Rice. II. Grain Yield Components. Genetics.

[15] Li Z.K., Luo L.J., Mei H.W., Wang D.L., Shu Q.Y., Tabien R., Zhong D.B., Ying C.S., Stansel J.W., Khush G.S. (2001). Overdominant Epistatic Loci Are the Primary Genetic Basis of Inbreeding Depression and Heterosis in Rice. I. Biomass and Grain Yield. Genetics.

[16] Malinovskaya L.P., Tishakova K.V., Bikchurina T.I., Slobodchikova A.Y., Torgunakov N.Y., Torgasheva A.A., Tsepilov Y.A., Volkova N.A., Borodin P.M. (2021). Negative Heterosis for Meiotic Recombination rate in Spermatocytes of the Domestic Chicken Gallus Gallus. Vavilov J. Genet. Breed..

[17] Mai C., Wen C., Xu Z., Xu G., Chen S., Zheng J., Sun C., Yang N. (2021). Genetic Basis of Negative Heterosis for Growth Traits in Chickens Revealed by Genome-Wide Gene Expression Pattern Analysis. J. Anim. Sci. Biotechnol..

[18] Arthur P.F., Makarechian M., Price M.A., Berg R.T. (1989). Heterosis, Maternal and Direct Effects in Double-Muscled and Normal Cattle: I. Reproduction and Growth Traits. J. Anim. Sci..

[19] Clasen J.B., Norberg E., Madsen P., Pedersen J., Kargo M. (2017). Estimation of Genetic Parameters and Heterosis for Longevity in Crossbred Danish Dairy Cattle. J. Dairy Sci..

[20] Iolchiev B.S., Volkova N.A., Bagirov V.A., Zinovieva N.A. (2020). Identification of Interspecific Hybrids Argali (Ovis Ammon) and Domestic Sheep (Ovis Aries) of Different Generations by Exterior Indicators. Sel’skokhozyaistvennaya Biol..

[21] Chang C.C., Chow C.C., Tellier L.C.A.M., Vattikuti S., Purcell S.M., Lee J.J. (2015). Second-Generation PLINK: Rising to the Challenge of Larger and Richer Datasets. Gigascience.

[22] Aulchenko Y.S., Ripke S., Isaacs A., van Duijn C.M. (2007). GenABEL: An R Library for Genome-Wide Association Analysis. Bioinformatics.

[23] Alderson G.L.H. (1999). The Development of a System of Linear Measurements to Provide an Assessment of Type and Function of Beef Cattle. Anim. Genet. Resour. Inf..

[24] Salako A.E. (2006). Application of Morphological Indices in the Assessment of Type and Function in Sheep. Int. J. Morphol..

[25] Dahl A., Iotchkova V., Baud A., Johansson A., Gyllensten U., Soranzo N., Mott R., Kranis A., Marchini J. (2016). A Multiple-Phenotype Imputation Method for Genetic Studies. Nat. Genet..

[26] Svishcheva G.R., Axenovich T.I., Belonogova N.M., van Duijn C.M., Aulchenko Y.S. (2012). Rapid Variance Components—Based Method for Whole-Genome Association Analysis. Nat. Genet..

[27] Zlobin A.S., Volkova N.A., Borodin P.M., Aksenovich T.I., Tsepilov Y.A. (2019). Recent Advances in Understanding Genetic Variants Associated with Growth, Carcass and Meat Productivity Traits in Sheep (Ovis Aries): An Update. Arch. Anim. Breed..

[28] McLaren W., Gil L., Hunt S.E., Riat H.S., Ritchie G.R.S., Thormann A., Flicek P., Cunningham F. (2016). The Ensembl Variant Effect Predictor. Genome Biol..

[29] Yang J., Lee S.H., Goddard M.E., Visscher P.M. (2011). GCTA: A Tool for Genome-Wide Complex Trait Analysis. Am. J. Hum. Genet..

[30] Zlobin A.S., Nikulin P.S., Volkova N.A., Zinovieva N.A., Iolchiev B.S., Bagirov V.A., Borodin P.M., Aksenovich T.I., Tsepilov Y.A. (2021). Multivariate Analysis Identifies Eight Novel Loci Associated with Meat Productivity Traits in Sheep. Genes.

[31] Chen P.W., Jian X., Yoon H.Y., Randazzo P.A. (2013). ARAP2 Signals through Arf6 and Rac1 to Control Focal Adhesion Morphology. J. Biol. Chem..

[32] Chen P.W., Luo R., Jian X., Randazzo P.A. (2014). The Arf6 GTPase-Activating Proteins ARAP2 and ACAP1 Define Distinct Endosomal Compartments That Regulate Integrin A5β1 Traffic. J. Biol. Chem..

[33] Chaudhari A., Håversen L., Mobini R., Andersson L., Ståhlman M., Lu E., Rutberg M., Fogelstrand P., Ekroos K., Mardinoglu A. (2016). ARAP2 Promotes GLUT1-Mediated Basal Glucose Uptake through Regulation of Sphingolipid Metabolism. Biochim. Biophys. Acta-Mol. Cell Biol. Lipids.

[34] De Souza T.C., da Cruz V.A.R., Mourão G.B., Pedrosa V.B., Rovadoscki G.A., Coutinho L.L., de Camargo G.M.F., Costa R.B., de Carvalho G.G.P., Pinto L.F.B. (2022). Estimates of Heritability and Candidate Genes for Primal Cuts and Dressing Percentage in Santa Ines Sheep. Livest. Sci..

[35] Lu X., Abdalla I.M., Nazar M., Fan Y., Zhang Z., Wu X., Xu T., Yang Z. (2021). Genome-Wide Association Study on Reproduction-Related Body-Shape Traits of Chinese Holstein Cows. Animals.

[36] Lee S.H., van der Werf J., Lee S.H., Lim D.J., Park E.W., Gondro C., Yoon D., Oh S.J., Kim O.H., Gibson J. (2012). Genome wide QTL mapping to identify candidate genes for carcass traits in Hanwoo (Korean Cattle). Genes Genom..

[37] Salpietro V., Perez-Dueñas B., Nakashima K., San Antonio-Arce V., Manole A., Efthymiou S., Vandrovcova J., Bettencourt C., Mencacci N.E., Klein C. (2018). A Homozygous Loss-of-function Mutation in PDE2A Associated to Early-onset Hereditary Chorea. Mov. Disord..

[38] Doummar D., Dentel C., Lyautey R., Metreau J., Keren B., Drouot N., Malherbe L., Bouilleret V., Courraud J., Valenti-Hirsch M.P. (2020). Biallelic PDE2A Variants: A New Cause of Syndromic Paroxysmal Dyskinesia. Eur. J. Hum. Genet..

[39] Miura K., Jacques K.M., Stauffer S., Kubosaki A., Zhu K., Hirsch D.S., Resau J., Zheng Y., Randazzo P.A. (2002). ARAP1: A Point of Convergence for Arf and Rho Signaling. Mol. Cell.

[40] Kulzer J.R., Stitzel M.L., Morken M.A., Huyghe J.R., Fuchsberger C., Kuusisto J., Laakso M., Boehnke M., Collins F.S., Mohlke K.L. (2014). A Common Functional Regulatory Variant at a Type 2 Diabetes Locus Upregulates ARAP1 Expression in the Pancreatic Beta Cell. Am. J. Hum. Genet..

[41] Ahmed Z.M., Riazuddin S., Bernstein S.L., Ahmed Z., Khan S., Griffith A.J., Morell R.J., Friedman T.B., Riazuddin S., Wilcox E.R. (2001). Mutations of the Protocadherin Gene PCDH15 Cause Usher Syndrome Type 1F. Am. J. Hum. Genet..

[42] Brown D., Swan A.A., Gill J.S., Ball A.J., Banks R.G. (2015). Genetic Parameters for Liveweight, Wool and Worm Resistance Traits in Multi-Breed Australian Meat Sheep. 1. Description of Traits, Fixed Effects, Variance Components and Their Ratios. Anim. Prod. Sci..

[43] Vargas Jurado N., Notter D.R., Taylor J.B., Brown D.J., Mousel M.R., Lewis R.M. (2022). Model Definition for Genetic Evaluation of Purebred and Crossbred Lambs Including Heterosis. J. Anim. Sci..

[44] Cloete S.W.P., Thutwa K., Scholtz A.J., Cloete J.J.E., Dzama K., Gilmour A.R., van Wyk J.B. (2021). Breed Effects and Heterosis for Weight Traits and Tick Count in a Cross between an Indigenous Fat-Tailed and a Commercial Sheep Breed. Trop. Anim. Health Prod..

