# Supplementary material for: Loci Associated with Negative Heterosis for Viability and Meat Productivity in Interspecific Sheep Hybrids
Source: Animals (Basel). 2023 Jan 3;13(1):184. doi: 10.3390/ani13010184 (PMC9817718; doi:10.3390/ani13010184)
Supplement: Supplementary file 1 [file animals-13-00184-s001.zip › animals-2023729-Figures.pdf]

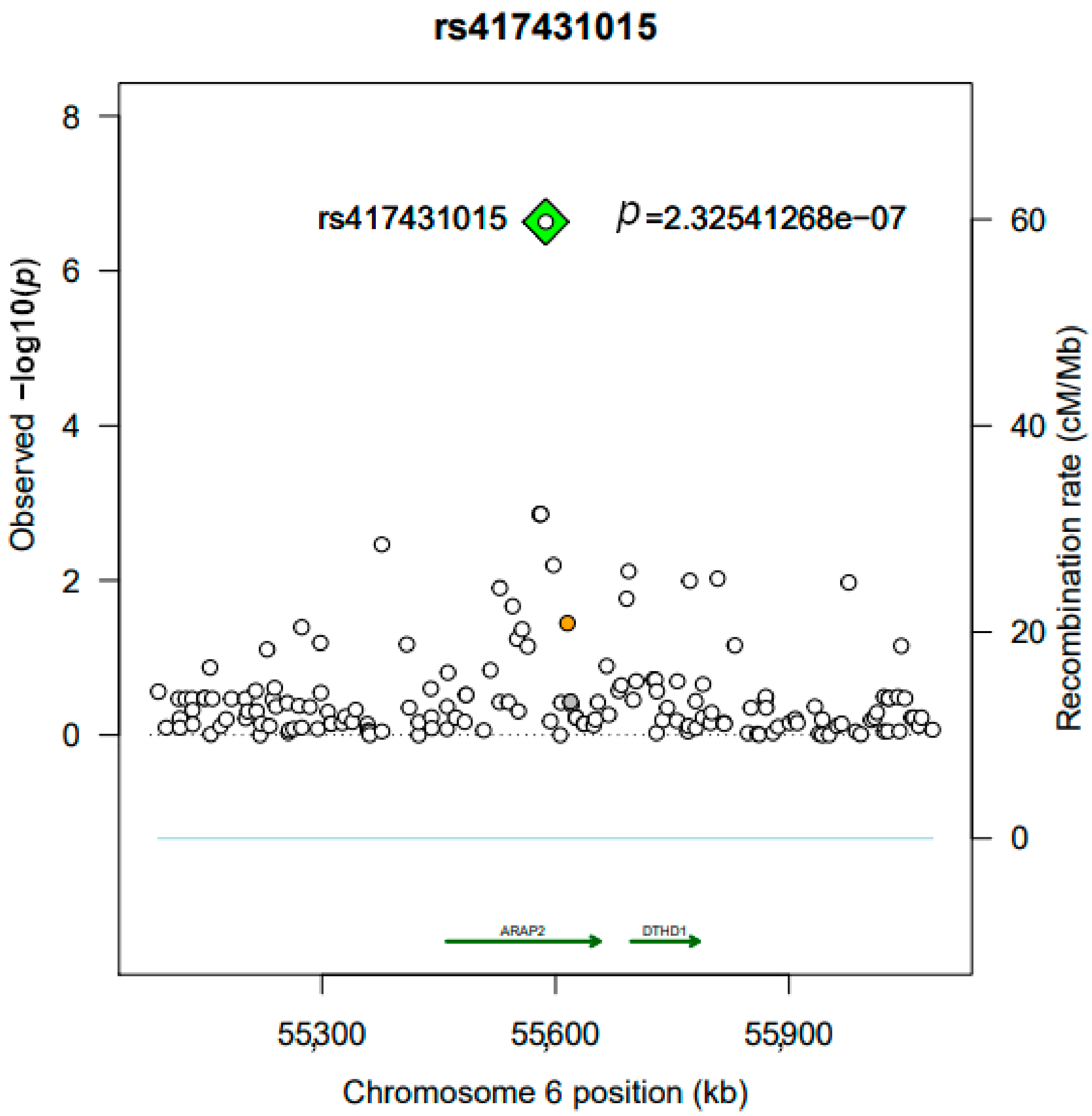

Supplementary Figure S1. The regional association plot for rs417431015.

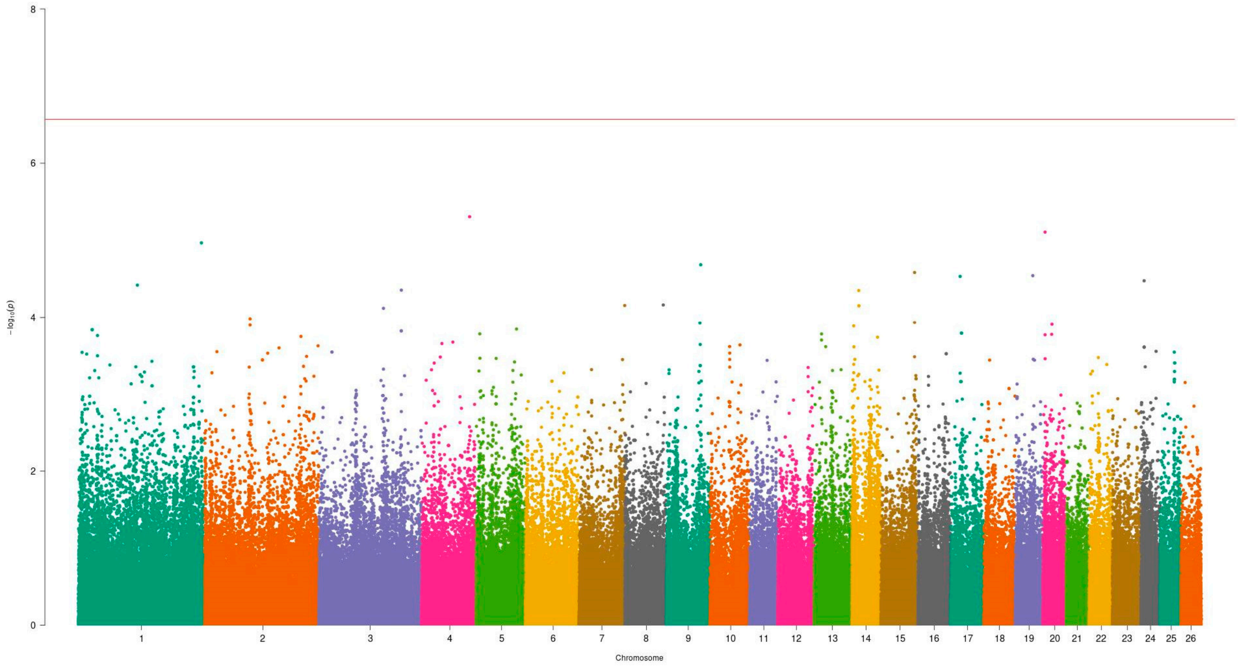

Supplementary Figure S2. The Manhattan plot for shortlegged index.

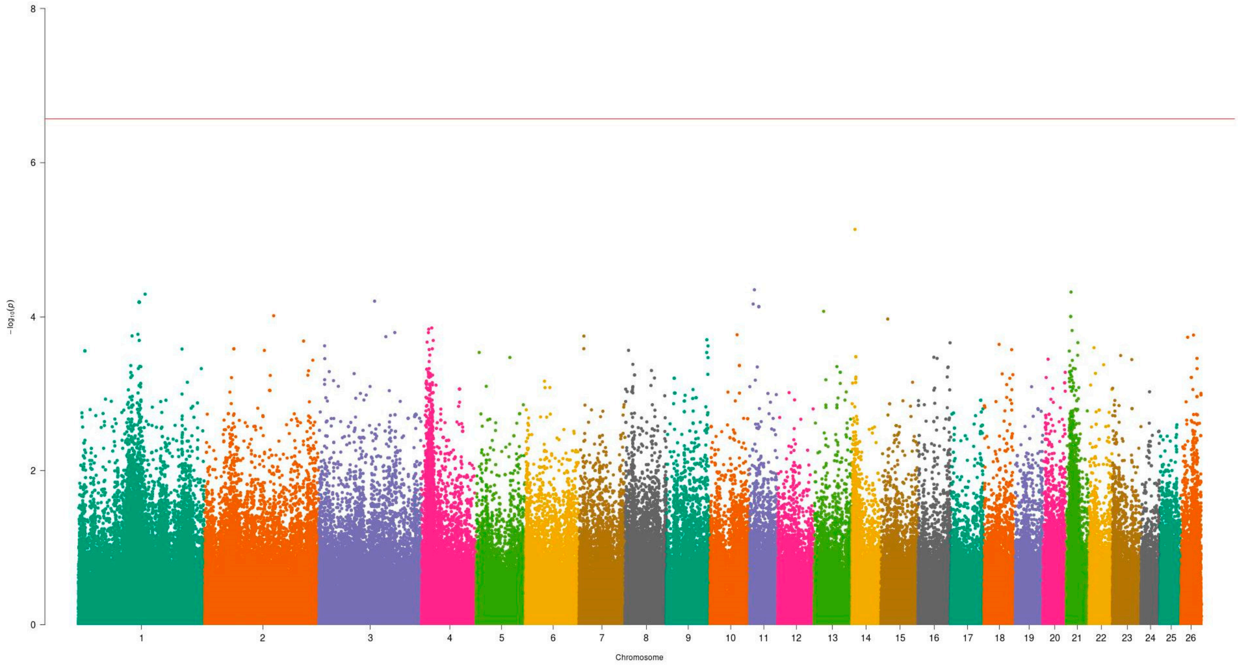

Supplementary Figure S3. The Manhattan plot for length index.

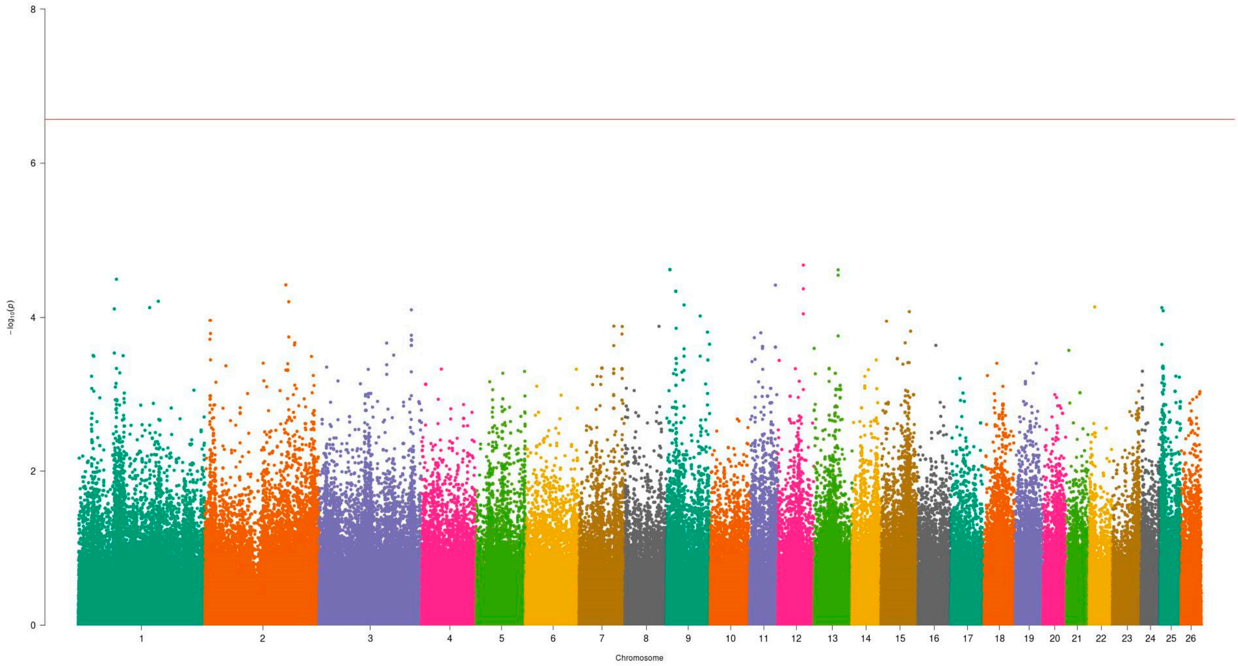

Supplementary Figure S4. The Manhattan plot for mass.

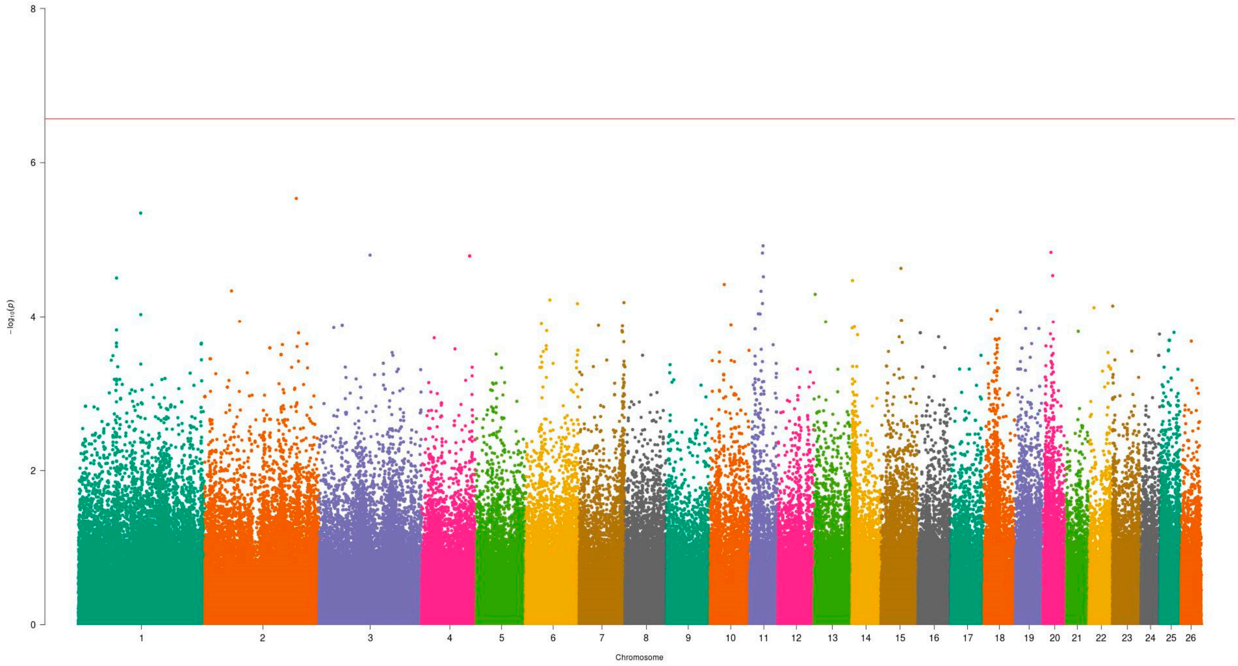

Supplementary Figure S5. The Manhattan plot for cummulative index.

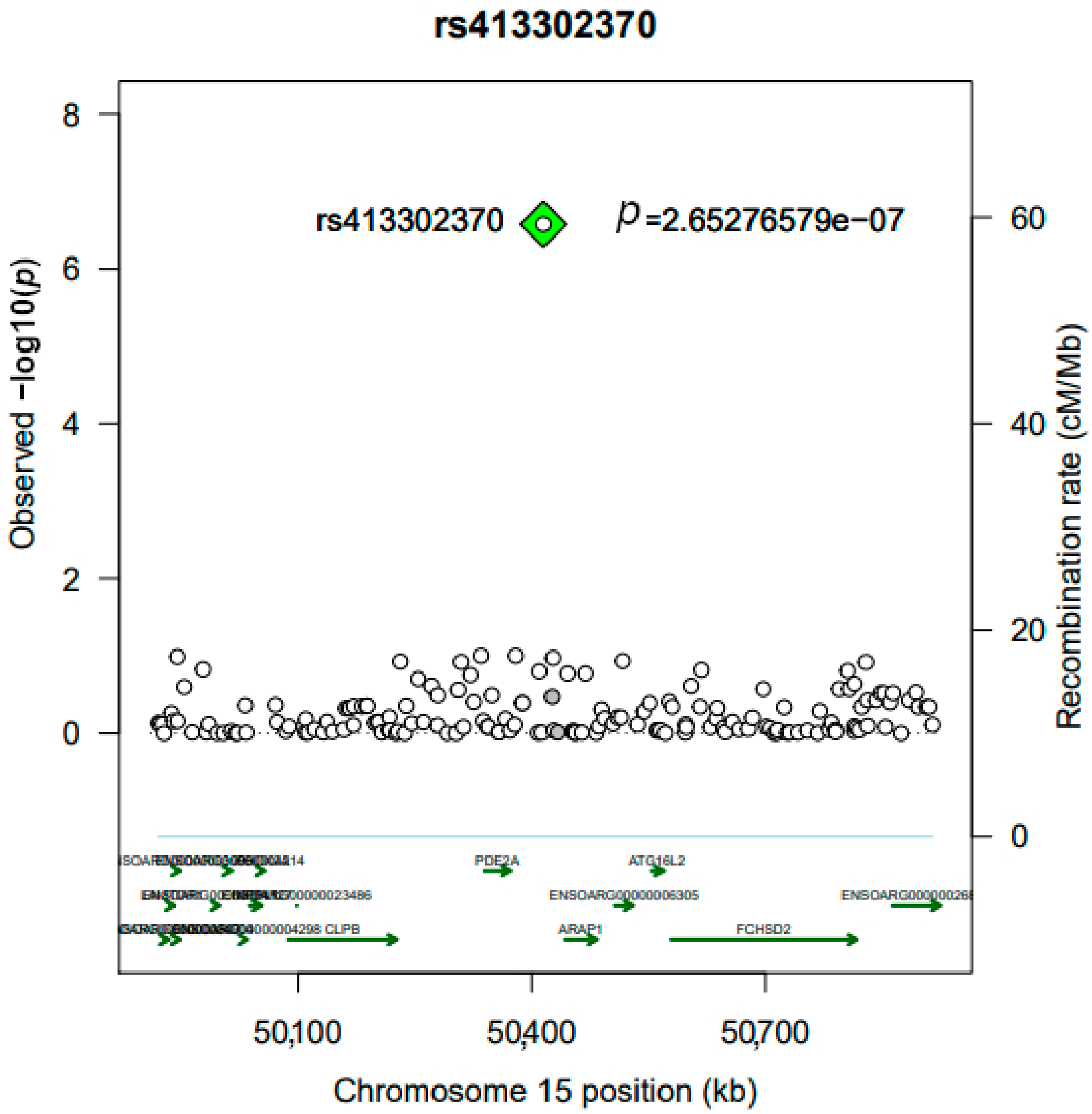

Supplementary Figure S6. The regional association plot for rs413302370.

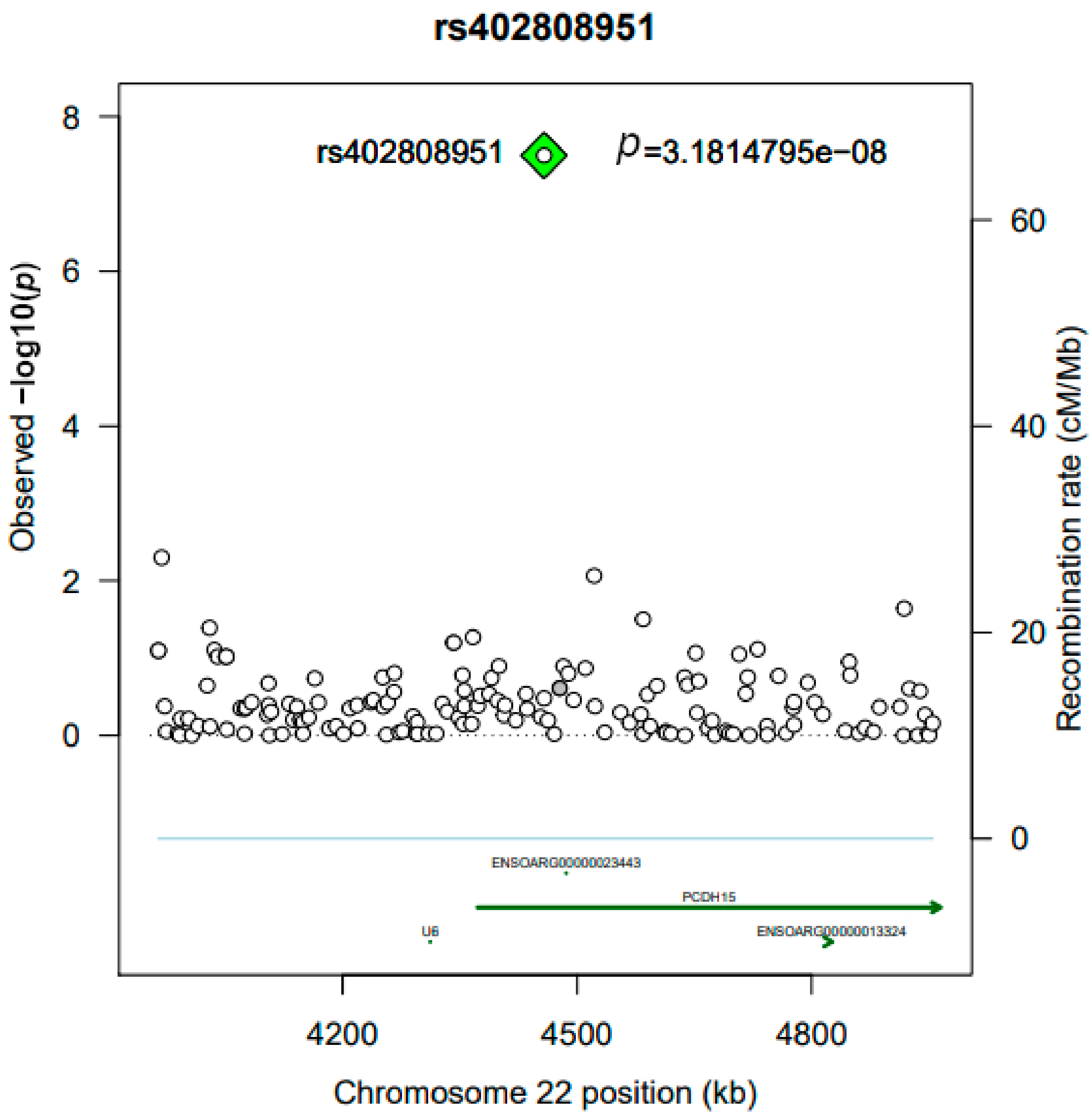

Supplementary Figure S7. The regional association plot for rs402808951.
